# Mitochondrial DNA Copy Number Raises the Potential of Left Frontopolar Hemodynamic Response as a Diagnostic Marker for Distinguishing Bipolar Disorder From Major Depressive Disorder

**DOI:** 10.3389/fpsyt.2019.00312

**Published:** 2019-05-08

**Authors:** Noa Tsujii, Ikuo Otsuka, Satoshi Okazaki, Masaya Yanagi, Shusuke Numata, Naruhisa Yamaki, Yoshihiro Kawakubo, Osamu Shirakawa, Akitoyo Hishimoto

**Affiliations:** ^1^Department of Neuropsychiatry, Kindai University Faculty of Medicine, Osaka-sayama, Japan; ^2^Department of Psychiatry, Kobe University Graduate School of Medicine, Kobe, Japan; ^3^Department of Psychiatry, Institute of Biomedical Science, Tokushima University Graduate School, Tokushima, Japan

**Keywords:** bipolar disorder, major depressive disorder, hemodynamics, mitochondria, near-infrared spectroscopy

## Abstract

**Background:** Given a lack of markers, diagnoses of bipolar disorder (BD) and major depressive disorder (MDD) rely on clinical assessment of symptoms. However, the depressive mood states of BD and depressive symptoms of MDD are often difficult to distinguish, which leads to misdiagnoses, which in turn leads to inadequate treatment. Previous studies have shown that the hemodynamic responses of the left frontopolar cortex measured by near-infrared spectroscopy (NIRS) differ between BD and MDD; these hemodynamic responses are associated with altered mitochondrial metabolism; and mitochondrial DNA copy number (mtDNAcn), an index of mitochondrial dysfunction, tends to decrease in BD and increase in MDD patients. In this study, we confirmed that mtDNAcn trends in opposite directions in BD and MDD. We then determined whether mtDNAcn could enhance the utility of NIRS as a diagnostic marker to distinguish between BD and MDD.

**Methods:** We determined mtDNAcn in peripheral blood samples from 58 healthy controls, 79 patients with BD, and 44 patients with MDD. Regional hemodynamic responses during a verbal fluency task (VFT) in 24 BD patients and 44 MDD patients, matched by age and depression severity, were monitored using NIRS.

**Results:** Measurements of mtDNAcn were lower in BD patients and higher in MDD patients than in controls. The left frontopolar region exhibited the most significant differences in mean VFT-related oxy-Hb changes between the BD and MDD groups. Multivariate logistic regression analysis with variables including age, sex, hemodynamic response of the left frontopolar region, and mtDNAcn showed high accuracy for distinguishing BD from MDD (area under the curve = 0.917; 95% confidence interval = 0.849–0.985). For the BD group, we observed a positive correlation between hemodynamic responses in the left frontopolar region and mtDNAcn, while for the MDD group, we observed a negative correlation.

**Conclusions:** Our findings suggest that the association between hemodynamic response and mitochondrial dysfunction in BD or MDD plays an important role in differentiating the pathophysiological mechanisms of BD from those of MDD.

## Introduction

Bipolar disorder (BD) and major depressive disorder (MDD) are severe mental disorders that cause long-term disability. Diagnoses of both diseases rely on clinical assessment of symptoms, due to the lack of available molecular or brain-imaging diagnostic tests available; however, the depressive mood state of BD and depressive symptoms of MDD are often confused with each other, which could lead to inadequate medical treatment (1).

As a diagnostic test for these disorders, near-infrared spectroscopy (NIRS) has gained increasing interest. NIRS is a noninvasive method for recording brain electrical activity by focal increases in cerebral blood flow (2). Previous NIRS studies have reported differential hemodynamic responses, primarily in the left frontopolar cortex, during verbal fluency tasks (VFTs) between BD patients, MDD patients, and healthy controls (3–7), consistent with findings from other neuroimaging techniques, including functional magnetic resonance imaging (fMRI) (8, 9). A multicenter, collaborative NIRS study reported that left frontal hemodynamic responses during VFTs were diagnostic for MDD with 75% accuracy and BD with 77% accuracy (10), although some skepticism regarding the diagnostic accuracy of NIRS, particularly the overdiagnosis of BD relative to MDD, remains (11). Considering the clinical need, diagnostic markers for distinguishing between BD and MDD may be much more valuable than markers for diagnosing BD or MDD relative to controls.

Previous physiological studies have reported that hemodynamic responses are associated with the metabolism of mitochondrial chromophores or with the cytochrome c oxidase redox state (2, 12). Accumulating evidence supports a role for mitochondrial dysfunction in pathophysiological differences between psychiatric disorders (13). BD has been associated with decreased mitochondrial electron transport chain activity and increased oxidative stress; moreover, multiple studies have reported an association between BD and the presence of mitochondrial DNA (mtDNA) variants (14). The mtDNA copy number, or mtDNAcn, has been used as an index of mitochondrial dysfunction and investigated in many diseases, including mood disorders (15–17). Recently, our meta-analysis showed that mtDNAcn was lower in BD patients than in controls in an Asian population (18). Furthermore, a large-scale study demonstrated that mtDNAcn was higher in MDD patients than in controls (19). These results suggest that mtDNAcn exhibits converse trends in BD and MDD; however, to date no single study has investigated these differences in BD and MDD simultaneously.

Therefore, in this study, we first confirmed that mtDNAcn exhibits converse trends in BD and MDD, and then determined whether mtDNAcn enhances the utility of NIRS as a marker to distinguish between BD and MDD.

## Materials and Methods

### Participants

For mtDNAcn measurement, patients who were clinically diagnosed with BD (n = 79, mean age ± standard deviation = 43.4 ± 12.4 years), patients with MDD (n = 44, 42.9 ± 10.8 years), and healthy controls (CON; n = 58, 48.2 ± 13.1 years) from the Osaka, Kobe, and Tokushima city areas of Japan (Hondo) (6, 18, 20, 21) were recruited between April 2013 and March 2017. All patients participated in this study *via* outpatient consultations. The gender distributions for the BD, MDD, and CON patient groups were (male/female) 40/39, 31/13, and 29/29, respectively. Diagnoses of BD or MDD were made using the criteria of DSM-IV or DSM-5.

Patients diagnosed with BD not otherwise specified (BD-NOS) and mixed features were excluded. Other exclusion criteria included a history of head trauma with loss of consciousness, current or previous neurological diseases, current or previous endocrine diseases, alcohol/substance abuse or addiction within the past 12 months, a history of electroconvulsive therapy, and comorbid anxiety disorder. Healthy controls were required to have no present, past, or first-degree relative history of psychiatric disorders, or any other current serious medical disorder. This study complied with the Declaration of Helsinki and was approved by the Ethics Committee of each institute. A portion of the data generated and analyzed during this study was included in our previous article (18).

Regional hemodynamic responses during a VFT in 24 patients with BD and 44 with MDD (matched by age and depression severity) were monitored by NIRS (**Table 1**). All 68 patients were diagnosed using the Japanese version of the Mini International Neuropsychiatric Interview (22) with DSM. All were diagnosed to be in a euthymic or depressive state on the day of scanning. Among the BD patients, 14 exhibited bipolar I disorder and 10 exhibited bipolar II disorder. Percentages of depressed and euthymic patients were 58% (14) and 42% (10) of the BD group, and 59% (26) and 40% (18) of the MDD group, respectively.

**Table 1:** Demographics of patients with bipolar disorder and patients with major depressive disorder.

	Patients with BD (n = 24)	Patients with MDD (n = 44)	χ^2^/t/U	*P* value
Age (years)	40.2 ± 9.3	42.9 ± 10.8	t = 1.05	0.30
Females (*n*, %)	12 (50.0)	13 (30)	χ^2^ = 2.80	0.10
Estimated IQ^a^	108.3 ± 8.8	105.8 ± 8.4	U = 446.0	0.36
Duration of illness (years)	11.4 ± 6.3	9.0 ± 8.2	U = 371.0	0.04
Antidepressant user (*n*, %) Mean daily dose (mg)^b^	11 (46) 90.6 ± 61.2	30 (68) 140.6 ± 76.7	χ^2^ = 3.24	0.07
Antipsychotic user (*n*, %) Mean daily dose (mg)^b^	11 (46) 176.1 ± 148.2	10 (23) 128.0 ± 85.0	χ^2^ = 3.88	0.05
Mood stabilizer user (*n*, %)	12 (50)	8 (18)	χ^2^ = 7.57	<0.01
Mean daily dose (mg)^b^				
- Lithium	510 ± 181	533 ± 149		
- Sodium valproate	600 ± 424	300 ± 0.0		
- Carbamazepine	300 ± 0.0	−		
- Lamotrigine	81.3 ± 48.0	50 ± 0.0		
HDRS_17_	8.6 ± 6.1	9.8 ± 6.5	U = 473.5	0.48
YMRS^a^	2.3 ± 2.8	0.5 ± 1.2	U = 322.0	<0.01
GAF	57.8 ± 14.9	61.6 ± 13.6	U = 431.0	0.21
VFT performances	16.0 ± 4.2	15.4 ± 5.6	t = 0.45	0.65
mtDNAcn	0.71 ± 0.36	0.92 ± 0.42	U = 3490	0.02

BD, bipolar disorder; GAF, global assessment of functioning; HDRS, Hamilton Depression Rating Scale; MDD, major depressive disorder; mtDNAcn, mitochondrial DNA copy number; VFT, verbal fluency task; YMRS, Young mania rating scale.

a Data were missing for one MDD patient.

b Mean daily dose was calculated only for users for antidepressants, antipsychotics, and each mood stabilizer, respectively.

### Assessment of Symptoms

Severity of depression was evaluated using the 17-item Hamilton Depression Rating Scale (HDRS_17_), administrated using a structured interview guide (23). Manic symptoms were assessed using the Young Mania Rating Scale (YMRS) (24). All patients with BD had a YMRS total score of 8 or less. Global functioning was assessed with the Global Assessment of Functioning (GAF) (21). IQ was estimated using the Japanese version of the National Adult Reading Test (25). Daily doses of all antidepressants and antipsychotics were converted to equivalent doses of imipramine and chlorpromazine, respectively (26).

### Measurement of mtDNAcn

DNA was extracted from peripheral whole blood samples using the QIAamp DNA Blood Midi Kit (Qiagen Inc., Valencia, CA), and quantified using a NanoDrop spectrophotometer (Thermo Scientific, Wilmington, DE). The mtDNAcn was calculated by measuring the amount of mtDNA (NADH dehydrogenase, subunit 1 [*ND1*]) relative to that of the nuclear gene *HGB* as previously described (27). All qPCR experiments were performed using a 7500 Real-Time PCR System (Applied Biosystems, Foster City, CA), with SYBR Green Master Mix (Applied Biosystems, Foster City, CA). The forward and reverse primer sequences and cycling conditions for the *ND1* gene were 5’-AAC ATA CCC ATG GCC AAC CT and 5’-AGC GAA GGG TTG TAG TAG CCC, respectively, with an initial heating step of 95°C for 10 min, followed by 40 cycles of 95°C for 15 s, 58°C for 20 s, and 72°C for 20 s. The forward and reverse primer sequences and cycling conditions for the *HGB* gene were 5’-GCT TCT GAC ACA ACT GTG TTC ACT AGC and 5’-CAC CAA CTT CAT CCA CGT TCA CC, respectively, with an initial heating step of 95°C for 10 min, followed by 40 cycles of 95°C for 15 s, 58°C for 20 s, and 72°C for 20 s. Each sample was amplified in triplicate, using 10 ng of DNA. Amplification of *ND1* and the single-copy gene *HGB* was performed in separate runs, using the same reference sample in the same well positions. A standard curve from a five-point serial-dilution series with reference DNA was constructed. Laboratory personnel who performed the assays were blinded to the identities and group memberships of the patients; moreover, all demographic data, as well as the sample order, were randomized in each batch of amplifications.

### Near-Infrared Spectroscopy

**Figure 1:**
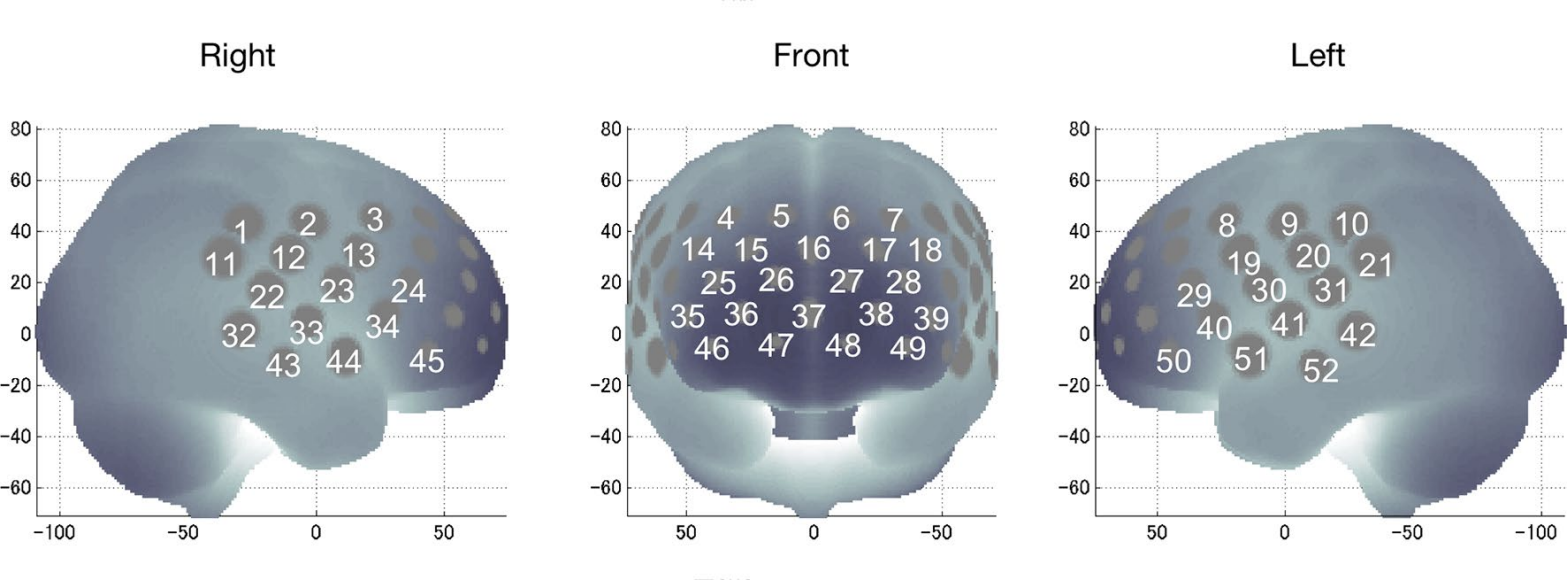
Locations of near-infrared spectroscopy channels for recording functional hemodynamics in the frontotemporal area (dorsolateral prefrontal, ventrolateral prefrontal, frontopolar, and temporal areas).

We used a 52-channel NIRS device (ETG-4000 Optical Topography System; Hitachi Medical Co., Tokyo, Japan) to measure changes in regional cortical Hb concentration in frontotemporal regions during cognitive activation as described previously (28, 29). Probes (17 emitters and 16 detectors, alternating) were fixed using 3 × 11 thermoplastic shells with an inter-optode distance of 3.0 cm. Each adjoining pair of an emitter and detector was referred to as a “channel,” resulting in 52 channels in total (**Figure 1**). The lowermost probes were positioned along the Fp1–Fp2 line, according to the International 10–20 system. The probes measure Hb values bilaterally from the prefrontal and temporal surface regions, at a depth of 20–30 mm from the scalp, which corresponds roughly to the depth of the surface of the cerebral cortex.

NIRS measures relative changes in oxy- and deoxy-Hb concentrations using two wavelengths (695 and 830 nm) of near-infrared light, based on the modified Beer–Lambert law (30). However, NIRS cannot measure the absolute path length from the emitter to the detector. We therefore recorded relative mean changes in Hb concentration from the baseline in mM·mm. The time resolution of signal acquisition was 0.1 s. Mean changes in task-related oxy-Hb levels were calculated by a linear fit to two baseline periods: the final 10 s of the pre-task period and the final 5 s of the post-task period (integral mode). We set the moving average window to 5 s to remove high-frequency noise from sources including heartbeats and small movements. Data from some channels were excluded for an excessive level of artifacts using a computer algorithm described previously (31); thus, the number of available channels varied among individuals, but mean numbers of channels did not differ between the groups [BD: mean ± SD, 49.7 ± 3.4; and MDD: mean ± SD, 49.4 ± 3.2; Mann–Whitney U test, U = 499.0, p = 0.70].

The spatial information for each channel was estimated using data from the Functional Brain Science Laboratory at the Jichi Medical University, Japan (32–34). The locations of NIRS channels in frontotemporal regions were estimated and anatomically labeled in the Chris Rorden MRIcro region of highest probability.

### Activation Task

Changes in hemoglobin levels were stimulated using VFTs because previous studies have shown measurable prefrontal activation during VFTs in healthy subjects (4, 29). The task procedure was similar to that described previously (28, 29). VFTs used in the present study included a 30-s pre-task baseline period, a 60-s task period comprising three 20-s blocks, and a 70-s post-task baseline period. During the pre- and post-task baseline periods, the subjects were instructed to repeat a train of syllables (“a, i, u, e, and o”). During the 60-s task, subjects were asked to generate as many words as possible that begin with that syllable. The possible syllables were: block 1 (0–20 s), “a,” “to,” or “na”; block 2 (20–40 s), “i,” “ki,” or “se”; and block 3 (40–60 s), “o,” “ta,” or “ha.” The number of correct words represented the subject’s performance score.

### Statistical Analysis

Demographic and clinical variables were compared between the study groups using a χ^2^ test for categorical variables and a *t*-test or Mann–Whitney U-test for continuous variables. The threshold for statistical significance was set at *P* < 0.05 (two-tailed). Regression analyses, performed using generalized linear models, a gamma distribution, and a log link, were used as needed to determine whether mtDNAcn variations between the patient groups were associated with covariates such as patient age and sex. Dummy variables [phenotype, control = 0, case (BD or MDD) = 1; sex, male = 1 and female = 2] were used where necessary.

To identify regional differences between the groups in hemodynamic responses of the 52 channels, mean oxy-Hb changes with normal distribution were compared using a *t*-test or the changes with non-normal distribution were compared using Mann–Whitney U-test with a Bonferroni-corrected *P*-value of <0.05 (i.e., 0.05/52; *P* = 0.001). Channel 38, which showed the most significant differences between BD and MDD for mean VFT-related oxy-Hb changes, and mtDNAcn were further examined as candidate biomarkers for distinguishing BD from MDD, using multivariate logistic regression analysis. Area under the receiver operating characteristic (ROC) curve analyses were used to measure discriminatory power. To test the relationships between mean oxy-Hb changes and demographic and clinical variables, we calculated Spearman correlation coefficients, again with a Bonferroni-corrected *P*-value of <0.05 (i.e., 0.05/52; *P* = 0.001).

All statistical tests were performed using IBM SPSS Statistics (version 22.0; IBM Corporation, Armonk, NY, USA) and R (version 3.4.1; The R Foundation for Statistical Computing, Vienna, Austria) software.

## Results

### mtDNAcn Differences Among Bipolar Disorder, Major Depressive Disorder, and Healthy Control Groups

**Figure 2:**
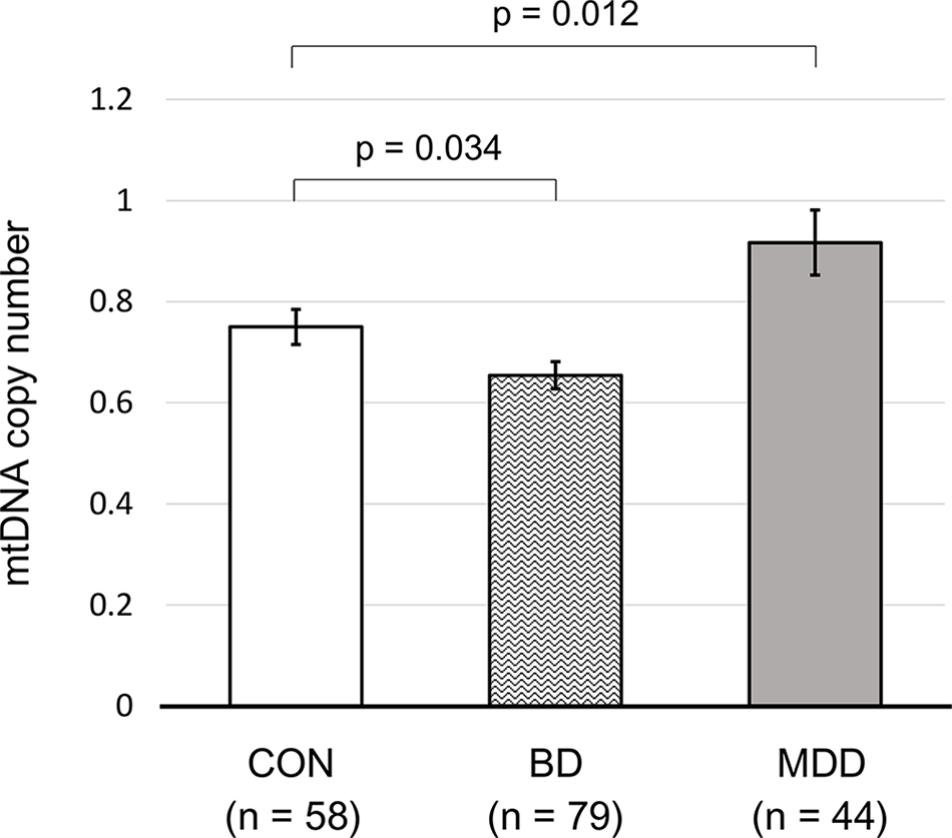
The mtDNA copy numbers of control subjects and patients with bipolar or major depressive disorder. All *P* values were calculated using generalized linear models, considering patient age and sex. Error bars represent the standard error of the mean. CON, control; BD, bipolar disorder; MDD, major depressive disorder.

Our regression analysis after adjusting for age and sex showed that patients with BD had a significantly lower mtDNAcn (β = −0.014, *P* = 0.034) than that of healthy controls; in contrast, patients with MDD had a significantly higher mtDNAcn than that of healthy controls (β = 0.108, *P* = 0.012; **Figure 2**, **Supplementary Table 1**).

### Demographics for Near-Infrared Spectroscopy Study

Compared with the MDD group, the BD group had a significantly longer duration of illness (*P* = 0.043), as well as larger ratios of antipsychotic usage (*P* = 0.049) and mood stabilizer usage (*P* = 0.006). In clinical variables, the BD group had higher scores on the YMRS, compared with the MDD group (*P* = 0.001), whereas group differences were not observed in VFT performance (*P* = 0.651). **Table 1** summarizes the demographic and clinical characteristics of the study groups.

### Near-Infrared Spectroscopy Data

#### Oxy-Hb Measurements in Bipolar Disorder and Major Depressive Disorder Groups

**Figure 3:**
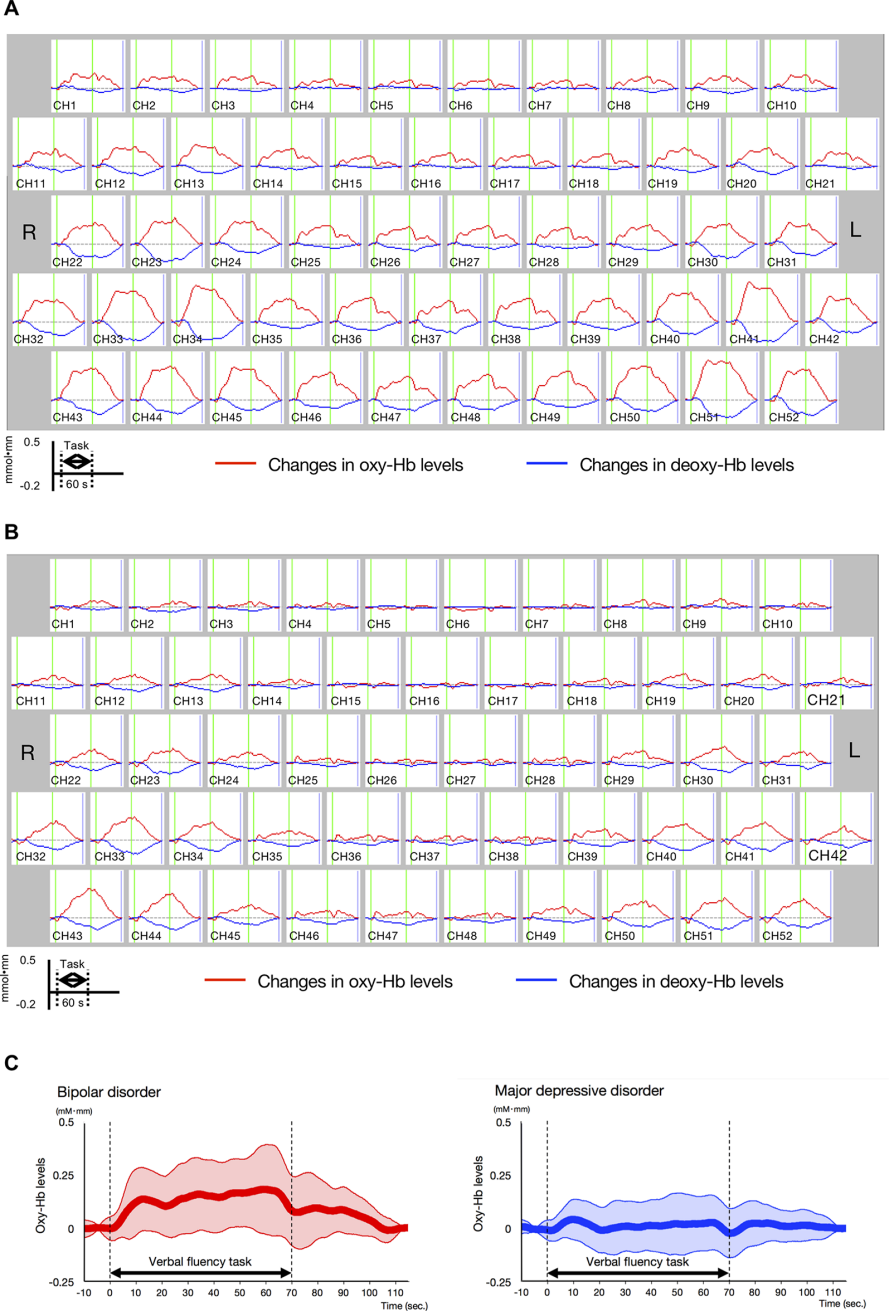
Time-course changes in oxygenated hemoglobin and deoxygenated hemoglobin levels in bipolar disorder (BD) and major depressive disorder (MDD). **(A)** Time-course changes in BD. **(B)** Time-course changes in MDD. In the BD group, oxy-Hb levels gradually increased after the start of the task period and decreased gradually in the post-task period mainly in the prefrontal channels, while the oxy-Hb levels in the MDD group were lower than those in the BD group in frontotemporal channels across the task period. **(C)** Differential time-course changes in oxy-Hb levels between BD and MDD groups, for a channel 38, in which a significant difference in VFT-related oxy-Hb levels was observed between study groups. Standard deviations are shown as pale red (BD) and blue-dotted (MDD) areas. The oxy-Hb levels of the BD group gradually increased after the start of the task period, while these of the MDD group were smaller across the task period.

Differences in oxy-Hb levels over time between the BD and MDD patient groups were clearly observed from the middle to the end of the VFT (**Figure 3A and B**; for details, see **Supplementary Figure 1**).

#### Differences in Mean Oxy-Hb Changes between Bipolar Disorder and Major Depressive Disorder Groups

The BD group exhibited significantly larger mean VFT-related oxy-Hb changes than the MDD group in four channels (ch17, ch27, ch38, and ch48; Bonferroni-corrected *P* < 0.001; **Table 2** **,** **Supplementary Table 2**). Among these four channels, ch38 exhibited the most significant difference in mean VFT-related oxy-Hb changes between the BD and MDD groups (t = 4.39). **Figure 3C** shows differential time-course changes in oxy-Hb levels between groups for a representative channel, ch38, in the left frontopolar region. We found no significant differences between the BD subjects with (n = 4) and without (n = 20) medication (**Supplementary Table 3**) and between the MDD subjects with (n = 11) and without (n = 32) medication (**Supplementary Table 4**).

**Table 2:** Comparison of VFT-related oxy-Hb changes of patients with bipolar disorder and those with major depressive disorder threshold, with Bonferroni-corrected *P* < 0.00096.

			MNI coordinate						
Estimated region	R/L	NIRS Ch	x	y	z	BD	MDD	t-value	*P*-value
Frontopolar region	L	Ch17	−22	57	32	0.07 ± 0.10	−0.01 ± 0.07	3.647	0.00053
	L	Ch27	−13	68	20	0.10 ± 0.12	0.01 ± 0.08	3.824	0.00029
	L	Ch38	−24	68	9	0.13 ± 0.013	0.01 ± 0.09	4.392	0.00004
	L	Ch48	−13	72	−3	0.13 ± 0.016	0.01 ± 0.10	3.657	0.00053

BD, bipolar disorder; Ch, channel; MDD, major depressive disorder; VFT, verbal fluency task.

#### Lack of Correlations between Oxy-Hb Changes and Clinical Variables

We did not find any other significant correlations between mean VFT-related oxy-Hb changes for each channel and any clinical variables (age, sex, estimated IQ, duration of illness, or dosage of antidepressants, antipsychotics or each drug of mood stabilizers, HDRS17, YMRS, GAF) in either patient group.

#### Multivariate Logistic Regression Analysis

**Figure 4:**
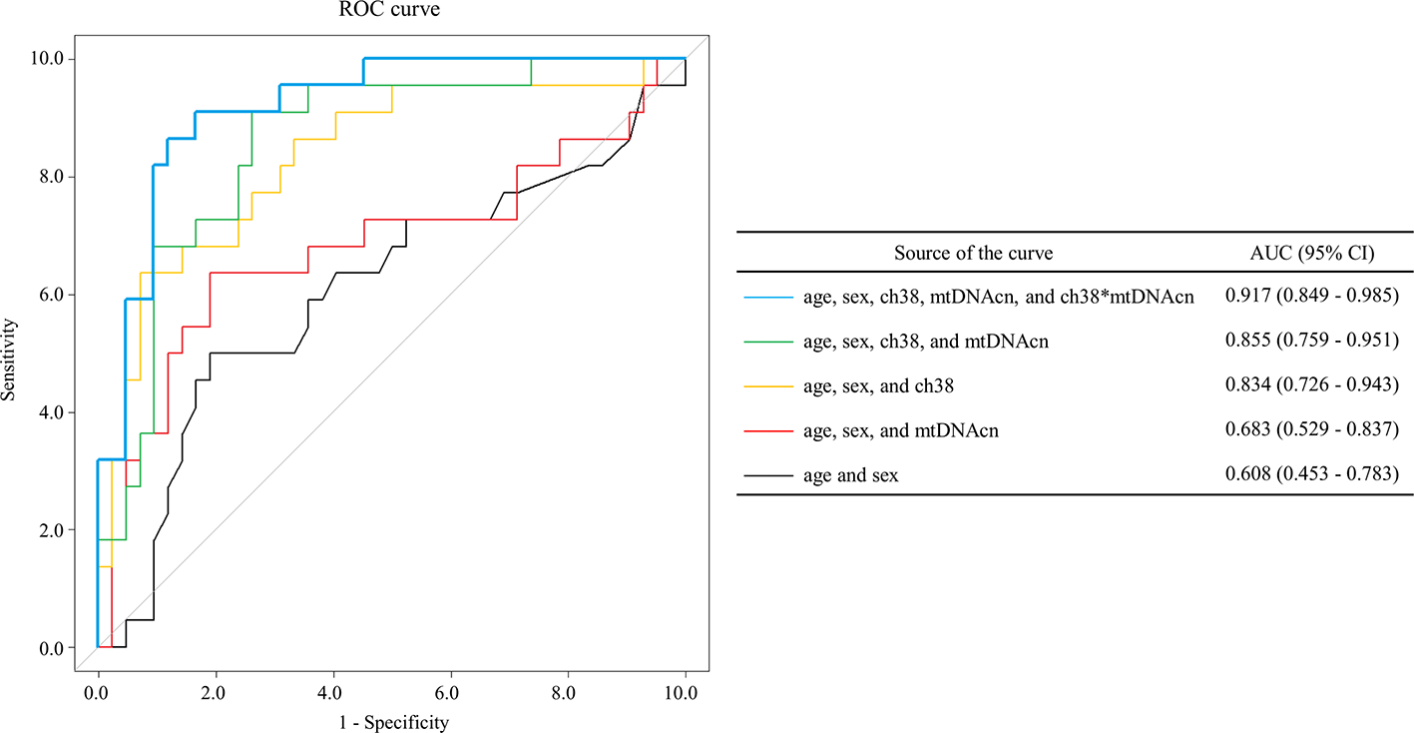
The receiver operating characteristic curves from multivariate logistic regression models. Multivariate logistic regression analysis [the response variable: phenotype (bipolar disorder, BD/major depressive disorder, MDD) using the explanatory variables age, sex, VFT-related oxy-Hb changes for channel 38, mitochondrial DNA copy number (mtDNAcn), and interaction between mean VFT-related oxy-Hb changes of ch38 and mtDNAcn] showed high accuracy [area under the curve (AUC) = 0.917 (95% CI = 0.849–0.985)].

Multivariate logistic regression analysis was performed to determine whether mean VFT-related oxy-Hb changes in the channels, combined with mtDNAcn, age, and sex, could discriminate BD from MDD. On first review, the correlation analyses and scatter plots showed a significant linear relationship among all the four channels (**Supplementary Figure 2**). We selected ch38 because it showed the most significant difference in mean VFT-related oxy-Hb changes between BD and MDD (**Table 2**); moreover, ch38 had a strong association with each of the other four channels. We performed a multivariate logistic regression analysis in which phenotype (BD/MDD) was the response variable, with age, sex, mean VFT-related oxy-Hb changes of ch38, mtDNAcn, and interaction between mean VFT-related oxy-Hb changes for ch38 and mtDNAcn as the explanatory variables (**Table 3**). The resulting model showed high accuracy, with an area under the curve (AUC) of 0.917 [95% CI = 0.849–0.985] (**Figure 4**). We also tested the models with age and sex, and/or mean VFT-related oxy-Hb changes of ch38, and/or mtDNAcn without the interaction. These models were less accurate, with AUCs ranging from 0.608 to 0.855 (**Figure 4**).

**Table 3:** Multivariate logistic regression analysis of bipolar disorder (BD)/major depressive disorder (MDD) with ch38 and mitochondrial DNA copy number (mtDNAcn).

Variable	B^a^	SE	*P*-value	OR	95% CI		Percentage of correct classifications
					Lower	Upper	
Age	0.015	0.040	0.708	1.015	0.938	1.099	BD = 81.8% MDD = 90.5% Overall = 87.5%
Sex	1.866	0.817	0.022	6.465	1.303	32.081	
ch38	30.917	11.345	0.006	2.67.E+13	5.90.E+3	1.21.E+23	
mtDNAcn	−4.320	1.867	0.021	0.0133	0.000343	0.516	
ch38*mtDNAcn	65.295	25.814	0.011	2.28.E+28	2.42.E+6	2.14.E+50	
Constant	−3.239	2.345	0.167				

A multivariate logistic regression analysis was performed to examine the ability to discriminate BD from MDD. Phenotype (BD/MDD) was the response variable, and age, sex, ch38, mtDNAcn, and interaction between ch38 and mtDNAcn, which was calculated after centering with each average, were the explanatory variables. The model *χ* ^2^ test showed significance (*χ* ^2^ = 41.016, df = 5, P < 0.001), and the individual variables also showed significance (P < 0.05) except age and constant. Hosmer and Lemeshow test showed that the regression formula was adequate (P = 0.424). The percentage of correct classifications was 87.5%.

a B, unstandardized partial regression coefficient.

SE, standard error; OR, odds ratio; CI, confidence interval.

#### Differential Correlation between NIRS Data and mtDNAcn

**Figure 5:**
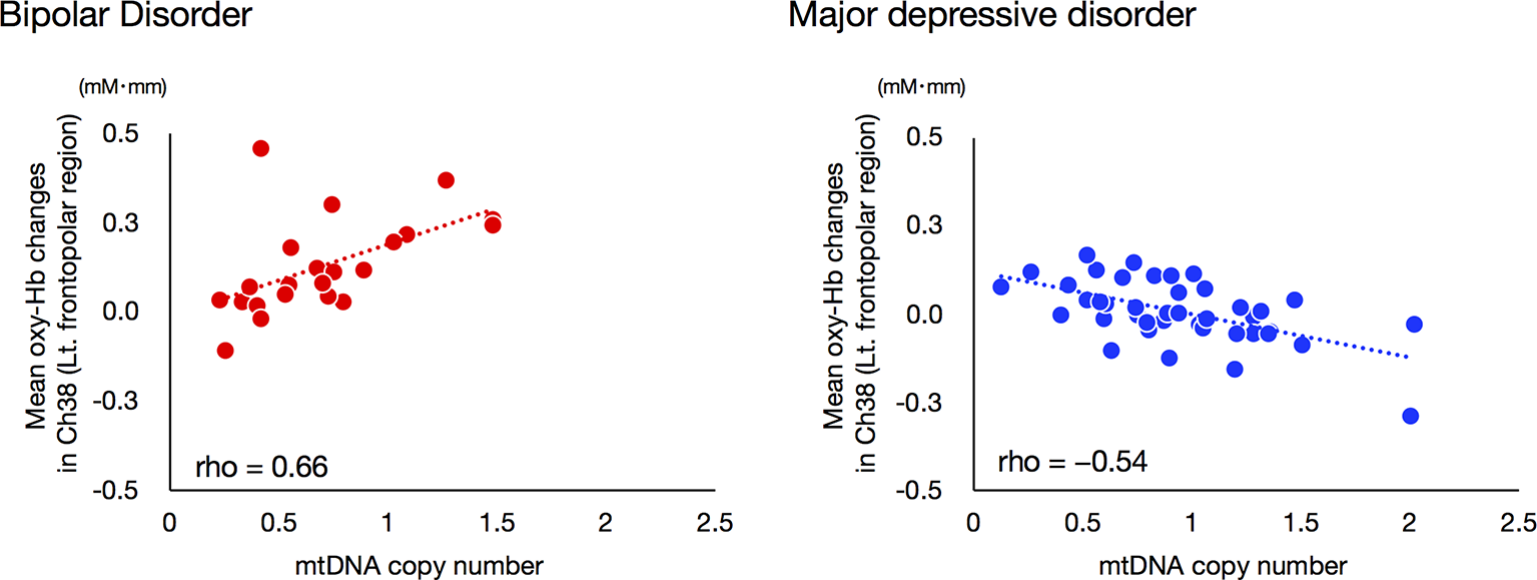
Relationships between mitochondrial DNA copy number and VFT-related oxy-Hb changes in bipolar disorder and major depressive disorder groups. A significant positive correlation between mtDNAcn and mean VFT-related oxy-Hb changes was found for the BD group, while a negative correlation was observed for the MDD group. The threshold for statistical significance was set at Bonferroni-corrected *P* < 0.00096.

We investigated the relationship between mean VFT-related oxy-Hb changes for ch38 and mtDNAcn, as the interaction between these two variables had a strong effect on ROC analysis. Interestingly, the mtDNAcn of the BD group was significantly positively correlated with mean VFT-related oxy-Hb changes in ch38 (rho = 0.660, Bonferroni-corrected *P* < 0.00096), while that of the MDD group was significantly negatively correlated (rho = −0.541, Bonferroni-corrected *P* < 0.00096; **Figure 5**).

## Discussion

This study is the first to investigate the relationship of changes in mtDNAcn in both BD and MDD simultaneously. In addition, this is also the first NIRS study to combine hemodynamic response data with mtDNAcn data. First, we showed that BD is likely associated with a reduction of mitochondrial DNA content and that BD patients exhibit changes in mtDNAcn that contrast with those exhibited by MDD patients. Next, we confirmed that the NIRS and mtDNAcn data from this study were consistent with data from previous studies; the differences in mean VFT-related oxy-Hb changes between BD and MDD appeared most significant in the left frontopolar region. Thus, we performed ROC analyses using Oxy-Hb changes of the left frontopolar region and mtDNAcn in order to determine whether the addition of mtDNAcn data increases the efficacy of NIRS as a diagnostic marker for distinguishing between BD and MDD. Notably, our ROC analysis achieved an AUC value of 0.917, which is far greater than those from previous studies using only NIRS data (approximately 0.75). We hypothesize that this increase is at least partially derived from our observation that the direction of the relationships between the oxy-Hb changes of the left frontopolar region (ch38) and mtDNAcn in this study was opposite in BD (rho = 0.66) and MDD (rho = −0.54). On the other hand, the strong correlation we observed between hemodynamic responses and mtDNAcn was only visible in the left prefrontal cortex, not in other brain regions.

Consistent with our focus on the left frontopolar region, previous neuroimaging studies in BD subjects revealed abnormal cortical thickness (CT) and dysfunction, primarily in the left frontal cortex. A recent, large study by the Bipolar Disorder Working Group within the ENIGMA Consortium found that the left rostral middle frontal cortex was one of the regions in which BD subjects exhibited the greatest reduction in CT (35). Interestingly, an MRI study of both BD and MDD subjects found significantly thinner CT in the left rostral middle frontal and frontopolar cortex in BD, but not in MDD (36). Furthermore, a proton magnetic resonance spectroscopy ((1)H MRS) study detected abnormal cellular energy and phospholipid metabolism in the left dorsolateral prefrontal cortex of medication-free individuals with BD, suggesting the presence of mitochondrial dysfunction in the left prefrontal cortex (37). Another study hypothesized that the VFT used in this study requires three shifts in mindset in rapidly switching from one phoneme to another; therefore, this task might be more sensitive to left prefrontal lobe deficits than VFTs that require fewer shifts (38). This hypothesis is consistent with previous neuroimaging studies using fMRI (39) and NIRS (4). In this context, previous NIRS studies using VFTs found alterations of oxy-Hb changes in frontotemporal regions in MDD, BD, or schizophrenia patients, particularly in the left frontopolar region (4, 5, 31).

In quantitative mtDNA, we used regression analysis with age and sex as covariates because both age and sex are well known to affect mtDNAcn; there is a positive relationship between age and mtDNAcn, as well as a higher mtDNAcn in females than in males (40). Some previous studies have reported lower mtDNAcn in peripheral blood of BD patients (15) and higher in MDD patients (19), although conflicting results exist (16, 17, 41). Some studies have found an association between mtDNAcn in peripheral blood and mtDNAcn in brain tissues (42, 43). Further, Cataldo et al. found that the postmortem BD prefrontal cortex samples had reduced numbers of mitochondria (44). Thus, aberrant mtDNAcn changes are likely to occur not only in peripheral blood but also in the brains of BD and MDD patients.

Because the mechanisms of the differences between oxy-Hb changes of the left frontopolar region in BD and MDD and the biological importance of mtDNAcn increases and decreases in peripheral blood remain unclear, our findings may be difficult to interpret; this seems to be the most important limitation in this study. The VFT as an activation task for NIRS measurement is regarded as a measure of executive dysfunction requiring efficient organization of verbal retrieval and recall, as well as self-monitoring aspects of cognition, effortful self-initiation, and inhibition of responses when appropriate (45). These characteristics of the task may contribute to differences in activity of the frontopolar region between BD and MDD. Further studies combining data from other measurements, such as fMRI and MRS, are needed to better understand the association(s) between mitochondrial dysfunction and neural activities in BD and MDD.

There were other limitations to our study. First, we were unable to exclude some potential confounders known to affect NIRS or mtDNAcn, such as smoking status and lifestyle factors (46–48). Second, we used peripheral whole-blood samples, including mixed leukocytes and platelets, for our mtDNAcn quantitation. However, there is evidence that whole-blood measurements are comparable to measurements in isolated lymphocytes (49), suggesting that this is unlikely to be an issue. Nonetheless, for studying psychiatric disorders such as BD or MDD, brain tissues are the standard target tissue. Third, the effect of psychotropic drugs on NIRS data and mtDNAcn should be considered. Indeed, most patients in this study were taking medications. Some studies have shown no association of antidepressant and mood stabilizer dosages with NIRS data in the prefrontal cortex (4, 50). In our study, the presence/absence of medication had no statistical effect on hemodynamic response in all channels, and the dosages of antidepressants, antipsychotics, and mood stabilizers were not correlated with hemodynamic responses in any channels. Fourth, it still remains to be determined whether clinical variables including duration of illness, mood state, and past episodes affect NIRS measurements. Previous reports have shown that neither the duration of illness nor the difference in mood state (euthymic or depressed) had any effect on the NIRS data (6, 51–53). In this study, we found no significant correlation between duration of illness and oxy-Hb changes in either group of patients. Fifth, our cohort has a significant difference between the gender ratios of our BD and MDD groups, although some studies have found no gender differences in NIRS data, including in the left frontopolar region (54, 55). These suggest that confounding effects of these factors are unlikely. However, our future studies using NIRS should control for these clinical variables. Finally, our sample size was too small to draw conclusions, and did not include either healthy controls or independent replication cohort. In addition, the measurements of hemodynamic responses by NIRS were conducted only in a subset of the patients whose mtDNAcn was measured. We are deeply aware of the need to test whether the high AUC obtained in our ROC analysis can be replicated in a well-designed independent cohort.

In summary, we have shown that patients with BD have a lower mtDNAcn than controls; this contrasts with higher mtDNAcn in patients with MDD than controls. Our findings suggest that mtDNAcn may be useful as an additional diagnostic marker to add to hemodynamic response for distinguishing BD from MDD. Further, the directions of the relationship between the oxy-Hb changes of the left frontopolar region and mtDNAcn were opposite in BD and MDD. Our findings suggest that the association between hemodynamic response and mitochondrial dysfunction plays an important role in differentiating the pathophysiological mechanisms of BD from those of MDD.

## Ethics Statement

This study complied with the Declaration of Helsinki and was approved by the Ethics Committee of each institute (the Ethics committee of the Kindai University Faculty of Medicine, the Ethical Committee for Genetic Studies of Kobe University Graduate School of Medicine, and the institutional ethics committee of Tokushima University).

## Author Contributions

AH designed the study and wrote the protocol. NT and IO managed the literature searches and analyses. NT, MY, SN, and YK corrected the data. NT, IO, SO, and NY undertook the statistical analysis, and NT and IO wrote the first draft of the manuscript. AH critically revised the text for important intellectual content. OS supervised and financially supported the study. All authors contributed to and have approved the final manuscript.

## Funding

This work was partly supported by a Grant-in-Aid for Scientific Research from the Japan Society for the Promotion of Science (No. 16K10229 and No. 17H04249).

## Conflict of Interest Statement

The authors declare that the research was conducted in the absence of any commercial or financial relationships that could be construed as a potential conflict of interest.

## Abbreviations

ATP, adenosine-5’-triphosphate; AUC, area under the curve; BD, bipolar disorder; CI, confidence interval; CT, cortical thickness; deoxy-HB, deoxygenated hemoglobin; fMRI, functional magnetic resonance imaging; GAF, global assessment of functioning; HDRS, Hamilton Depression Rating Scale; MDD, major depressive disorder; mtDNAcn, mitochondrial DNA copy number; NIRS, near-infrared spectroscopy; oxy-Hb, oxygenated hemoglobin; OR, odds ratio; ROC, receiver operating characteristic; SE, standard error; VFT, verbal fluency task; YMRS, Young mania rating scale; (1)H MRS, proton magnetic resonance spectroscopy.
