# Supplementary material for: Mitochondrial DNA Copy Number Raises the Potential of Left Frontopolar Hemodynamic Response as a Diagnostic Marker for Distinguishing Bipolar Disorder From Major Depressive Disorder
Source: Front Psychiatry. 2019 May 8;10:312. doi: 10.3389/fpsyt.2019.00312 (PMC6518968; doi:10.3389/fpsyt.2019.00312)
Supplement: Supplementary file 1 [file Table_1.pdf]

Supplementary Material 1

Mitochondrial DNA copy number raises the potential of left frontopolar hemodynamic response as a diagnostic marker for distinguishing bipolar disorder from major depressive disorder

Noa Tsujii†, Ikuo Otsuka†, Satoshi Okazaki, Masaya Yanagi, Shusuke Numata, Naruhisa Yamaki, Yoshihiro Kawakubo, Osamu Shirakawa, Akitoyo Hishimoto*

† These authors contributed equally to this work.

*** Correspondence:** Akitoyo Hishimoto: hishipon@med.kobe-u.ac.jp

Supplementary Table 1. Regression analyses of mtDNA copy number in patients with major depressive or bipolar disorders and healthy control subjects.

	mtDNA copy number			
	β^{a}	s.e.	t	p value[b]
Total samples (n = 137); BD (n = 79) and CON (n = 58)				
Phenotype (BD vs. CON)	-0.014	0.063	-2.138	**0.034**
Age (years)	0.001	0.002	0.052	0.959
Sex (male vs. female)	-0.010	0.063	-0.165	0.869
Total samples (n = 102); MDD (n = 44) and CON (n = 58)				
Phenotype (MDD vs. CON)	0.108	0.042	2.578	**0.012**
Age (years)	0.004	0.003	1.208	0.230
Sex (male vs. female)	-0.005	0.085	-0.057	0.955

[a] β represents regression coefficients derived from generalized linear models.

[b] p values shown in bold are significant at < 0.05.

Abbreviations: BD, bipolar disorder; CON, control; MDD, major depressive disorder; s.e., standard error.
