# Supplementary material for: Mitochondrial DNA Copy Number Raises the Potential of Left Frontopolar Hemodynamic Response as a Diagnostic Marker for Distinguishing Bipolar Disorder From Major Depressive Disorder
Source: Front Psychiatry. 2019 May 8;10:312. doi: 10.3389/fpsyt.2019.00312 (PMC6518968; doi:10.3389/fpsyt.2019.00312)
Supplement: Supplementary file 2 [file Table_2.pdf]

Supplementary Material 3

Mitochondrial DNA copy number raises the potential of left frontopolar hemodynamic response as a diagnostic marker for distinguishing bipolar disorder from major depressive disorder

Noa Tsujii†, Ikuo Otsuka†, Satoshi Okazaki, Masaya Yanagi, Shusuke Numata, Naruhisa Yamaki, Yoshihiro Kawakubo, Osamu Shirakawa, Akitoyo Hishimoto*

† These authors contributed equally to this work.

*** Correspondence:** Akitoyo Hishimoto: hishipon@med.kobe-u.ac.jp

Supplementary Table 2. Comparison of VFT-related oxy-Hb changes in patients with bipolar disorder and those with major depressive disorder.

NIRS Ch	MINI coordinate*			BD		MDD		P-value
	x	y	z	Mean	SD	Mean	SD	
Ch01	64.9	-28.1	42.4	0.075	0.134	0.008	0.082	0.05832
Ch02	59.7	-3.2	42.5	0.072	0.124	0.007	0.103	0.05110
Ch03	47.8	22.5	44.1	0.071	0.109	0.014	0.077	0.08210
Ch04	32.6	40.8	43.7	0.044	0.075	0.013	0.054	0.19602
Ch05	12.6	52.0	44.6	0.037	0.083	-0.006	0.062	0.11446
Ch06	-10.5	52.2	44.5	0.048	0.103	-0.017	0.068	0.01066
Ch07	-30.4	41.2	43.6	0.043	0.107	-0.007	0.068	0.01556
Ch08	-46.0	23.3	43.9	0.054	0.089	0.022	0.076	0.11857
Ch09	-57.1	-1.3	42.8	0.057	0.111	0.022	0.106	0.27955
Ch10	-63.3	-25.4	42.2	0.063	0.097	0.000	0.090	0.01038

Ch11	68.4	-19.2	17.5	0.076	0.128	0.029	0.096	0.29265
Ch12	63.8	7.8	20.2	0.103	0.124	0.037	0.116	0.01257
Ch13	53.6	35.8	20.1	0.124	0.156	0.039	0.094	0.02276
Ch14	37.1	57.4	19.9	0.094	0.120	0.018	0.089	0.04392
Ch15	14.5	68.3	21.3	0.052	0.094	0.002	0.084	0.10214
Ch16	-12.8	67.8	20.1	0.063	0.100	-0.005	0.070	0.00176
Ch17	-35.1	57.6	20.3	0.066	0.100	-0.009	0.068	0.00053
Ch18	-51.7	36.3	19.1	0.058	0.110	0.024	0.067	0.12256
Ch19	-61.6	9.5	20.0	0.095	0.104	0.048	0.110	0.09445
Ch20	-67.3	-16.8	18.8	0.092	0.143	0.037	0.104	0.02601
Ch21	69.1	-13.1	-10.3	0.077	0.100	0.017	0.082	0.05326
Ch22	59.6	10.6	-8.3	0.097	0.127	0.039	0.147	0.06203
Ch23	52.9	42.9	-5.8	0.128	0.139	0.052	0.156	0.08000
Ch24	38.1	63.2	-4.2	0.129	0.162	0.044	0.101	0.03646
Ch25	14.8	70.8	-2.6	0.109	0.143	0.024	0.100	0.05110
Ch26	-12.8	71.5	-2.8	0.093	0.135	0.010	0.105	0.00726
Ch27	-35.1	63.4	-4.4	0.100	0.123	0.005	0.080	0.00029
Ch28	-50.9	44.5	-6.2	0.086	0.109	0.021	0.079	0.03210
Ch29	-57.4	14.3	-7.8	0.096	0.106	0.062	0.109	0.18695

Ch30	-68.3	-11.5	-12.0	0.108	0.148	0.066	0.144	0.30545
Ch31	67.1	-35.7	29.9	0.109	0.169	0.039	0.133	0.07414
Ch32	66.5	-10.5	31.0	0.127	0.147	0.061	0.125	0.10804
Ch33	57.7	16.4	31.4	0.174	0.175	0.102	0.153	0.08374
Ch34	44.4	40.9	32.1	0.193	0.243	0.085	0.121	0.05219
Ch35	24.4	57.5	32.2	0.133	0.148	0.046	0.113	0.00877
Ch36	2.1	60.3	32.0	0.140	0.158	0.023	0.127	0.01280
Ch37	-22.4	57.2	32.4	0.103	0.177	0.010	0.096	0.00722
Ch38	-42.0	41.9	31.7	0.131	0.135	0.009	0.088	0.00004
Ch39	-55.2	17.5	31.4	0.125	0.133	0.057	0.098	0.01896
Ch40	-64.5	-8.2	31.3	0.176	0.204	0.083	0.123	0.03626
Ch41	-66.4	-33.6	30.4	0.211	0.246	0.074	0.157	0.01269
Ch42	70.8	-29.1	2.2	0.130	0.172	0.034	0.143	0.02410
Ch43	65.9	-4.1	5.4	0.173	0.184	0.131	0.165	0.31038
Ch44	58.8	26.7	8.1	0.185	0.201	0.101	0.160	0.08207
Ch45	46.6	52.1	7.2	0.187	0.203	0.059	0.103	0.01922
Ch46	26.8	67.9	8.5	0.157	0.164	0.041	0.104	0.00926
Ch47	2.4	68.6	8.2	0.132	0.160	0.032	0.127	0.01077
Ch48	-23.9	68.1	8.5	0.134	0.156	0.015	0.102	0.00053

Ch49	-44.3	52.8	6.3	0.157	0.136	0.060	0.095	0.00134
Ch50	-56.6	28.1	7.1	0.199	0.184	0.082	0.157	0.00382
Ch51	-63.8	-1.5	6.1	0.241	0.261	0.088	0.164	0.00862
Ch52	-69.1	-27.5	1.4	0.162	0.231	0.068	0.156	0.15120

Abbreviations: BD, bipolar disorder; Ch, channel; MDD, major depressive disorder.

The threshold for statistical significance was set at Bonferroni-corrected $p < 0.00096$.

* The spatial information for each channel was estimated using data from the Functional Brain Science Laboratory at the Jichi Medical University, Japan [1-3].

References

1. Rorden, C., and Brett, M. (2000). Stereotaxic display of brain lesions. Behav Neurol 12, 191-200.

2. Singh, A.K., Okamoto, M., Dan, H., Jurcak, V., and Dan, I. (2005). Spatial registration of multichannel multi-subject fNIRS data to MNI space without MRI. Neuroimage 27, 842-851. doi: 10.1016/j.neuroimage.2005.05.019.

3. Tsuzuki, D., Jurcak, V., Singh, A.K., Okamoto, M., Watanabe, E., and Dan, I. (2007). Virtual spatial registration of stand-alone fNIRS data to MNI space. Neuroimage 34, 1506-1518. doi: 10.1016/j.neuroimage.2006.10.043.
