# Supplementary material for: Mitochondrial DNA Copy Number Raises the Potential of Left Frontopolar Hemodynamic Response as a Diagnostic Marker for Distinguishing Bipolar Disorder From Major Depressive Disorder
Source: Front Psychiatry. 2019 May 8;10:312. doi: 10.3389/fpsyt.2019.00312 (PMC6518968; doi:10.3389/fpsyt.2019.00312)
Supplement: Supplementary file 3 [file Table_3.pdf]

Supplementary Material 4

Mitochondrial DNA copy number raises the potential of left frontopolar hemodynamic response as a diagnostic marker for distinguishing bipolar disorder from major depressive disorder

Noa Tsujii†, Ikuo Otsuka†, Satoshi Okazaki, Masaya Yanagi, Shusuke Numata, Naruhisa Yamaki, Yoshihiro Kawakubo, Osamu Shirakawa, Akitoyo Hishimoto*

† These authors contributed equally to this work.

*** Correspondence:** Akitoyo Hishimoto: hishipon@med.kobe-u.ac.jp

Supplementary Table 3. Comparison of VFT-related oxy-Hb changes in bipolar-disorder patients with and without medication.

Abbreviations: BD, bipolar disorder; Ch, channel; MDD, major depressive disorder.

NIRS Ch	MINI coordinate*			BD without medication (n=4)		BD with medication (n=20)		P-value**
	x	y	z	Mean	SD	Mean	SD	
Ch01	64.9	-28.1	42.4	0.062	0.118	0.077	0.140	0.84495
Ch02	59.7	-3.2	42.5	0.093	0.098	0.067	0.131	0.34204
Ch03	47.8	22.5	44.1	0.057	0.100	0.075	0.113	0.83746
Ch04	32.6	40.8	43.7	-0.001	0.014	0.053	0.080	0.34707
Ch05	12.6	52.0	44.6	-0.007	0.019	0.045	0.088	0.52494
Ch06	-10.5	52.2	44.5	0.138	0.184	0.030	0.074	0.18257
Ch07	-30.4	41.2	43.6	0.041	0.203	0.043	0.086	1.00000
Ch08	-46.0	23.3	43.9	0.063	0.032	0.053	0.097	0.79409
Ch09	-57.1	-1.3	42.8	0.105	0.045	0.050	0.118	0.08701
Ch10	-63.3	-25.4	42.2	0.112	0.110	0.053	0.094	0.18257
Ch11	68.4	-19.2	17.5	0.036	0.068	0.083	0.137	0.62733
Ch12	63.8	7.8	20.2	0.054	0.082	0.113	0.131	0.50548
Ch13	53.6	35.8	20.1	0.103	0.096	0.128	0.168	0.61118
Ch14	37.1	57.4	19.9	0.067	0.170	0.099	0.113	0.57538
Ch15	14.5	68.3	21.3	0.005	0.043	0.061	0.100	0.73687
Ch16	-12.8	67.8	20.1	0.103	0.079	0.055	0.104	0.47713
Ch17	-35.1	57.6	20.3	0.089	0.158	0.062	0.089	1.00000
Ch18	-51.7	36.3	19.1	-0.007	0.166	0.071	0.097	0.62733

Ch19	-61.6	9.5	20.0	0.164	0.088	0.081	0.104	0.11462
Ch20	-67.3	-16.8	18.8	0.110	0.097	0.088	0.153	0.68154
Ch21	69.1	-13.1	-10.3	0.090	0.101	0.074	0.103	0.85187
Ch22	59.6	10.6	-8.3	0.077	0.106	0.101	0.132	0.92105
Ch23	52.9	42.9	-5.8	0.143	0.071	0.125	0.150	0.63364
Ch24	38.1	63.2	-4.2	0.111	0.115	0.133	0.172	1.00000
Ch25	14.8	70.8	-2.6	0.064	0.138	0.118	0.145	0.73687
Ch26	-12.8	71.5	-2.8	0.056	0.112	0.098	0.140	0.76341
Ch27	-35.1	63.4	-4.4	0.111	0.129	0.097	0.125	0.91097
Ch28	-50.9	44.5	-6.2	0.060	0.121	0.092	0.109	0.68154
Ch29	-57.4	14.3	-7.8	0.094	0.076	0.096	0.113	0.97007
Ch30	-68.3	-11.5	-12.0	0.239	0.131	0.085	0.141	0.14561
Ch31	67.1	-35.7	29.9	0.124	0.140	0.105	0.177	0.90683
Ch32	66.5	-10.5	31.0	0.149	0.090	0.122	0.159	0.65208
Ch33	57.7	16.4	31.4	0.097	0.078	0.193	0.189	0.33519
Ch34	44.4	40.9	32.1	0.158	0.051	0.201	0.267	0.45647
Ch35	24.4	57.5	32.2	0.093	0.179	0.141	0.145	0.79409
Ch36	2.1	60.3	32.0	0.106	0.080	0.143	0.165	0.95238
Ch37	-22.4	57.2	32.4	0.129	0.116	0.101	0.184	0.70130
Ch38	-42.0	41.9	31.7	0.118	0.009	0.133	0.142	0.77922
Ch39	-55.2	17.5	31.4	0.108	0.162	0.128	0.132	0.97007
Ch40	-64.5	-8.2	31.3	0.334	0.375	0.144	0.147	0.43121
Ch41	-66.4	-33.6	30.4	0.217	0.134	0.210	0.267	0.65208
Ch42	70.8	-29.1	2.2	0.096	0.226	0.137	0.165	1.00000
Ch43	65.9	-4.1	5.4	0.115	0.015	0.182	0.196	0.69791
Ch44	58.8	26.7	8.1	0.144	0.021	0.191	0.217	0.88722
Ch45	46.6	52.1	7.2	0.147	0.094	0.195	0.220	0.96872
Ch46	26.8	67.9	8.5	0.122	0.042	0.164	0.179	0.68154
Ch47	2.4	68.6	8.2	0.177	0.075	0.125	0.170	0.40429
Ch48	-23.9	68.1	8.5	0.172	0.064	0.130	0.162	0.55411
Ch49	-44.3	52.8	6.3	0.157	0.068	0.157	0.144	0.63467
Ch50	-56.6	28.1	7.1	0.288	0.219	0.181	0.178	0.15735
Ch51	-63.8	-1.5	6.1	0.325	0.330	0.221	0.250	0.57444
Ch52	-69.1	-27.5	1.4	0.109	0.224	0.174	0.238	0.63525

Abbreviations: BD, bipolar disorder; Ch, channel; MDD, major depressive disorder.

The threshold for statistical significance was set at Bonferroni-corrected $p < 0.00096$.

* The spatial information for each channel was estimated using data from the Functional Brain Science Laboratory at the Jichi Medical University, Japan [1-3].

**Mann-Whitney U test

References

1. Rorden, C., and Brett, M. (2000). Stereotaxic display of brain lesions. Behav Neurol 12, 191-200.

2. Singh, A.K., Okamoto, M., Dan, H., Jurcak, V., and Dan, I. (2005). Spatial registration of multichannel multi-subject fNIRS data to MNI space without MRI. Neuroimage 27, 842-851. doi: 10.1016/j.neuroimage.2005.05.019.

3. Tsuzuki, D., Jurcak, V., Singh, A.K., Okamoto, M., Watanabe, E., and Dan, I. (2007). Virtual spatial registration of stand-alone fNIRS data to MNI space. Neuroimage 34, 1506-1518. doi: 10.1016/j.neuroimage.2006.10.043.
