# Supplementary material for: Mitochondrial DNA Copy Number Raises the Potential of Left Frontopolar Hemodynamic Response as a Diagnostic Marker for Distinguishing Bipolar Disorder From Major Depressive Disorder
Source: Front Psychiatry. 2019 May 8;10:312. doi: 10.3389/fpsyt.2019.00312 (PMC6518968; doi:10.3389/fpsyt.2019.00312)
Supplement: Supplementary file 4 [file Table_4.pdf]

Supplementary Material 5

Mitochondrial DNA copy number raises the potential of left frontopolar hemodynamic response as a diagnostic marker for distinguishing bipolar disorder from major depressive disorder

Noa Tsujii†, Ikuo Otsuka†, Satoshi Okazaki, Masaya Yanagi, Shusuke Numata, Naruhisa Yamaki, Yoshihiro Kawakubo, Osamu Shirakawa, Akitoyo Hishimoto*

† These authors contributed equally to this work.

*** Correspondence:** Akitoyo Hishimoto: hishipon@med.kobe-u.ac.jp

Supplementary Table 4. Comparison of VFT-related oxy-Hb changes in patients with major depressive disorder with and without medication

Abbreviations: BD, bipolar disorder; Ch, channel; MDD, major depressive disorder.

NIRS Ch	MINI coordinate*			MDD without medication (n=11)		MDD with medication (n=33)		P-value**
	x	y	z	Mean	SD	Mean	SD	
Ch01	64.9	-28.1	42.4	0.033	0.086	0.000	0.080	0.20112
Ch02	59.7	-3.2	42.5	0.070	0.106	-0.016	0.094	0.01737
Ch03	47.8	22.5	44.1	0.069	0.062	-0.006	0.073	0.00711
Ch04	32.6	40.8	43.7	0.041	0.057	0.003	0.051	0.05076
Ch05	12.6	52.0	44.6	0.033	0.050	-0.019	0.061	0.01548
Ch06	-10.5	52.2	44.5	0.013	0.065	-0.026	0.068	0.16091
Ch07	-30.4	41.2	43.6	0.038	0.078	-0.020	0.059	0.06740
Ch08	-46.0	23.3	43.9	0.062	0.073	0.010	0.074	0.09885
Ch09	-57.1	-1.3	42.8	0.065	0.108	0.006	0.103	0.23724
Ch10	-63.3	-25.4	42.2	0.042	0.079	-0.014	0.090	0.08187
Ch11	68.4	-19.2	17.5	0.017	0.089	0.033	0.100	0.76890
Ch12	63.8	7.8	20.2	0.039	0.114	0.036	0.119	0.76890
Ch13	53.6	35.8	20.1	0.094	0.072	0.022	0.095	0.00743
Ch14	37.1	57.4	19.9	0.024	0.066	0.016	0.096	0.81005
Ch15	14.5	68.3	21.3	0.045	0.081	-0.013	0.081	0.05211
Ch16	-12.8	67.8	20.1	0.025	0.076	-0.015	0.066	0.13046
Ch17	-35.1	57.6	20.3	0.015	0.089	-0.017	0.059	0.25209
Ch18	-51.7	36.3	19.1	0.061	0.070	0.012	0.063	0.07368

Ch19	-61.6	9.5	20.0	0.064	0.095	0.042	0.116	0.55176
Ch20	-67.3	-16.8	18.8	0.048	0.113	0.034	0.102	0.61157
Ch21	69.1	-13.1	-10.3	0.058	0.059	0.002	0.085	0.03539
Ch22	59.6	10.6	-8.3	0.008	0.082	0.048	0.161	0.65572
Ch23	52.9	42.9	-5.8	0.077	0.143	0.045	0.161	0.51291
Ch24	38.1	63.2	-4.2	0.064	0.096	0.037	0.103	0.61150
Ch25	14.8	70.8	-2.6	0.055	0.089	0.014	0.103	0.30782
Ch26	-12.8	71.5	-2.8	0.052	0.106	-0.005	0.103	0.15285
Ch27	-35.1	63.4	-4.4	0.039	0.080	-0.006	0.078	0.13763
Ch28	-50.9	44.5	-6.2	0.062	0.080	0.007	0.074	0.06476
Ch29	-57.4	14.3	-7.8	0.085	0.070	0.055	0.119	0.26011
Ch30	-68.3	-11.5	-12.0	0.028	0.106	0.078	0.153	0.19563
Ch31	67.1	-35.7	29.9	0.053	0.085	0.035	0.146	0.68551
Ch32	66.5	-10.5	31.0	0.053	0.055	0.064	0.145	0.89000
Ch33	57.7	16.4	31.4	0.086	0.055	0.107	0.173	0.61136
Ch34	44.4	40.9	32.1	0.116	0.130	0.076	0.120	0.38836
Ch35	24.4	57.5	32.2	0.049	0.099	0.045	0.118	0.87637
Ch36	2.1	60.3	32.0	0.055	0.098	0.013	0.135	0.64958
Ch37	-22.4	57.2	32.4	0.060	0.095	-0.006	0.092	0.09318
Ch38	-42.0	41.9	31.7	0.038	0.058	0.001	0.093	0.28880
Ch39	-55.2	17.5	31.4	0.071	0.066	0.052	0.107	0.59283
Ch40	-64.5	-8.2	31.3	0.042	0.087	0.096	0.131	0.41388
Ch41	-66.4	-33.6	30.4	0.002	0.072	0.101	0.172	0.06160
Ch42	70.8	-29.1	2.2	0.047	0.107	0.031	0.153	0.92398
Ch43	65.9	-4.1	5.4	0.064	0.082	0.149	0.178	0.24222
Ch44	58.8	26.7	8.1	0.141	0.162	0.090	0.161	0.19883
Ch45	46.6	52.1	7.2	0.089	0.111	0.050	0.101	0.44366
Ch46	26.8	67.9	8.5	0.097	0.094	0.021	0.101	0.05954
Ch47	2.4	68.6	8.2	0.096	0.141	0.010	0.116	0.09246
Ch48	-23.9	68.1	8.5	0.051	0.075	0.003	0.108	0.16983
Ch49	-44.3	52.8	6.3	0.082	0.062	0.053	0.103	0.46310
Ch50	-56.6	28.1	7.1	0.085	0.116	0.081	0.171	0.75649
Ch51	-63.8	-1.5	6.1	0.035	0.104	0.106	0.178	0.27196
Ch52	-69.1	-27.5	1.4	0.027	0.120	0.082	0.165	0.32929

Abbreviations: BD, bipolar disorder; Ch, channel; MDD, major depressive disorder.

The threshold for statistical significance was set at Bonferroni-corrected $p < 0.00096$.

* The spatial information for each channel was estimated using data from the Functional Brain Science Laboratory at the Jichi Medical University, Japan [1-3].

**Mann-Whitney U test

References

1. Rorden, C., and Brett, M. (2000). Stereotaxic display of brain lesions. Behav Neurol 12, 191-200.

2. Singh, A.K., Okamoto, M., Dan, H., Jurcak, V., and Dan, I. (2005). Spatial registration of multichannel multi-subject fNIRS data to MNI space without MRI. Neuroimage 27, 842-851. doi: 10.1016/j.neuroimage.2005.05.019.

3. Tsuzuki, D., Jurcak, V., Singh, A.K., Okamoto, M., Watanabe, E., and Dan, I. (2007). Virtual spatial registration of stand-alone fNIRS data to MNI space. Neuroimage 34, 1506-1518. doi: 10.1016/j.neuroimage.2006.10.043.
