# Supplementary material for: Mitochondrial DNA Copy Number Raises the Potential of Left Frontopolar Hemodynamic Response as a Diagnostic Marker for Distinguishing Bipolar Disorder From Major Depressive Disorder
Source: Front Psychiatry. 2019 May 8;10:312. doi: 10.3389/fpsyt.2019.00312 (PMC6518968; doi:10.3389/fpsyt.2019.00312)
Supplement: Supplementary file 5 [file Image_1.pdf]

frontiers

Supplementary Material 2

Mitochondrial DNA copy number raises the potential of left frontopolar hemodynamic response as a diagnostic marker for distinguishing bipolar disorder from major depressive disorder

Noa Tsujii†, Ikuo Otsuka†, Satoshi Okazaki, Masaya Yanagi, Shusuke Numata, Naruhisa Yamaki, Yoshihiro Kawakubo, Osamu Shirakawa, Akitoyo Hishimoto*

† These authors contributed equally to this work.

*** Correspondence:** Akitoyo Hishimoto: hishipon@med.kobe-u.ac.jp

Supplementary Figure 1. t-value graphs of oxy-Hb signal comparisons between bipolar disorder (BD) and major depressive disorder (MDD) groups during verbal fluency tasks.

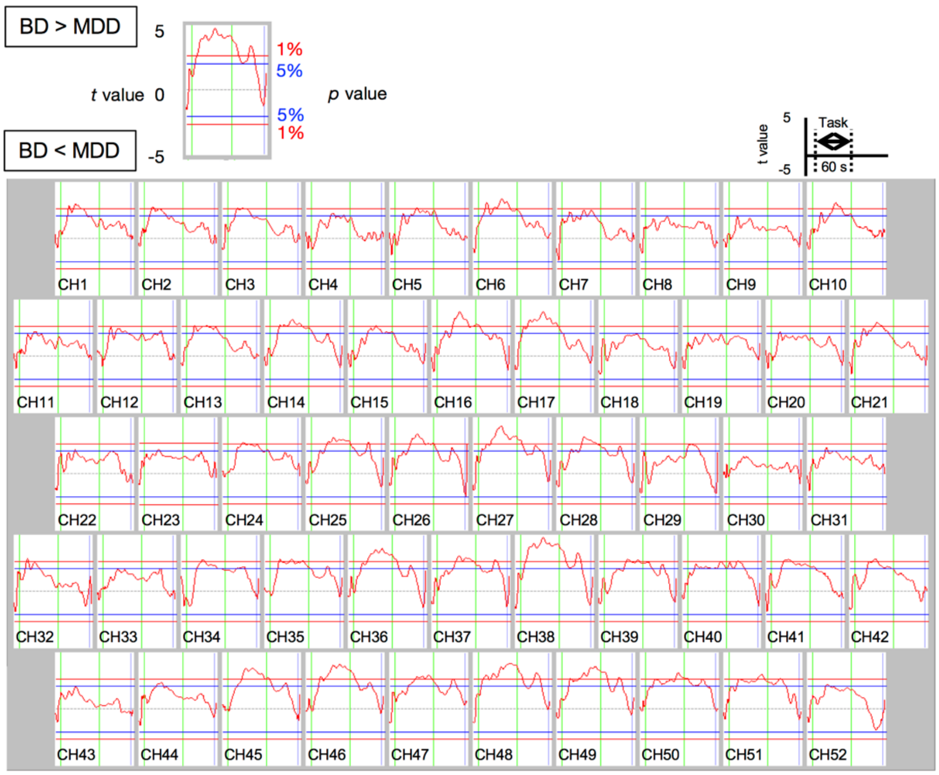

Supplementary Figure 1. Oxy-Hb increases in the BD group were significantly larger than these in the MDD group during the late period of VFT. The blue and red lines in each t graph correspond to

the statistical significance levels of 5% and 1%, respectively. Statistically significant differences between the BD and the MDD groups were observed middle to end of the task period mainly in the prefrontal channels. These results were consistent with previous findings by Kameyama et al. (2006).
